# iOntoBioethics: A Framework for the Agile Development of Bioethics Ontologies in Pandemics, Applied to COVID-19

**DOI:** 10.3389/fmed.2021.619978

**Published:** 2021-05-21

**Authors:** Mohammed Odeh, Faten F. Kharbat, Rana Yousef, Yousra Odeh, Dina Tbaishat, Nancy Hakooz, Rana Dajani, Asem Mansour

**Affiliations:** ^1^Cancer Care Informatics Programme, King Hussein Cancer Center (KHCC), Amman, Jordan; ^2^Faculty of Environment and Technology, University of the West of England, Bristol, United Kingdom; ^3^Software Engineering and Computer Science Department, College of Engineering, Al Ain University, Abu Dhabi, United Arab Emirates; ^4^Computer Information Systems Department, King Abdullah II School for Information Technology (KASIT), The University of Jordan, Amman, Jordan; ^5^Software Engineering Department, Faculty of Information Technology (FIT), Applied Science Private University, Amman, Jordan; ^6^Library and Information Science Department, University of Jordan, Amman, Jordan; ^7^Department of Biopharmaceutics and Clinical Pharmacy, School of Pharmacy, University of Jordan, Amman, Jordan; ^8^Department of Biology and Biotechnology, Hashemite University, Zarqa, Jordan; ^9^Jepson School of Leadership, University of Richmond, Richmond, VA, United States

**Keywords:** bioethics, COVID-19, pandemic, bioethics ontology, bioethics informatics, iOntoBioethics, agile framework, design science research methodology

## Abstract

**Background:** Few ontological attempts have been reported for conceptualizing the bioethics domain. In addition to limited scope representativeness and lack of robust methodological approaches in driving research design and evaluation of bioethics ontologies, no bioethics ontologies exist for pandemics and COVID-19. This research attempted to investigate whether studying the bioethics research literature, from the inception of bioethics research publications, facilitates developing highly agile, and representative computational bioethics ontology as a foundation for the automatic governance of bioethics processes in general and the COVID-19 pandemic in particular.

**Research Design:** The iOntoBioethics agile research framework adopted the Design Science Research Methodology. Using systematic literature mapping, the search space resulted in 26,170 Scopus indexed bioethics articles, published since 1971. iOntoBioethics underwent two distinctive stages: (1) Manually Constructing Bioethics (MCB) ontology from selected bioethics sources, and (2) Automatically generating bioethics ontological topic models with all 26,170 sources and using special-purpose developed Text Mining and Machine-Learning (TM&ML) engine. Bioethics domain experts validated these ontologies, and further extended to construct and validate the Bioethics COVID-19 Pandemic Ontology.

**Results:** Cross-validation of the MCB and TM&ML bioethics ontologies confirmed that the latter provided higher-level abstraction for bioethics entities with well-structured bioethics ontology class hierarchy compared to the MCB ontology. However, both bioethics ontologies were found to complement each other forming a highly comprehensive Bioethics Ontology with around 700 concepts and associations COVID-19 inclusive.

**Conclusion:** *The iOntoBioethics framework yielded the first* agile, semi-automatically generated, literature-based, and domain experts validated *General Bioethics* and *Bioethics Pandemic Ontologies Operable in COVID-19 context with readiness for automatic governance of bioethics processes*. These ontologies will be regularly and semi-automatically enriched as iOntoBioethics is proposed as an open platform for scientific and healthcare communities, in their infancy COVID-19 learning stage. iOntoBioethics not only it contributes to better understanding of bioethics processes, but also serves as a bridge linking these processes to healthcare systems. Such big data analytics platform has the potential to automatically inform bioethics governance adherence given the plethora of developing bioethics and COVID-19 pandemic knowledge. Finally, iOntoBioethics contributes toward setting the first building block for forming the field of “*Bioethics Informatics”*.

## Introduction

One of the key rationales behind developing machine interpretable ontologies is to resolve semantic heterogeneities between key concepts in a particular domain. Such an approach will facilitate common understanding and communication language between both humans and machine leading to better analysis and reusing of the underlying domain knowledge, along with the explicit representation and automatic reasoning based on related conceptual domain assumptions. In healthcare, for instance, context-aware systems must adapt to their changing dynamic environment. Ontology concepts are elicited and implemented in various healthcare computing systems (1). For example, ontology has been employed in the medical field to: enhance the functionality of complex medical data, provide informed medical prescriptions, and reduce errors in diagnosis (1). In addition, ontologies contribute to developing a global mental health ethics to serve the need of having autonomy-driven bioethics in non-western cultures (2). Furthermore, with the emergence of IoT and viable 5G networks, technology has been revolutionizing communication among healthcare systems. Therefore, the role of ontologies is considered a major building block in resolving semantic heterogeneities between healthcare systems in a global context (1).

The bioethics literature reports on few limited ontological attempts to conceptualize the bioethics domain with limited scope representativeness (3). In addition to a lack of a robust methodological approach in driving the research design and evaluation of resultant bioethics ontologies, the literature does not report on the existence of bioethics ontologies in pandemics and more specifically for COVID-19. Therefore, this research aims to develop an agile, highly representative, and robust ontological model within the domain of bioethics in general, and amidst pandemics in particular such as COVID-19. This is anticipated to achieve a better understanding of bioethics processes and automatic governance of these processes when linked to the respective information systems operating in healthcare centers, research and development institutions, civil society organizations, and businesses affected by bioethics.

Our main research hypothesis states that “investigating the bioethics research literature, from the inception of bioethics research publications, leads to identifying a highly agile representative set of bioethics conceptual entities, and governance relationships of bioethics processes”. A methodological research framework (iOntoBioethics) has been fit-for-purpose developed to prove this research hypothesis guided by the Design Science Research Methodology (DSRM) (4) and utilizing the systematic literature mapping method. The search space utilized more than 26,000 Scopus-indexed articles with emphasis on bioethics processes in order to inform whether a semi-automatically generated bioethics ontology is comparable to a manually developed generalized bioethics ontology developed also during the course of this research. The sufficiency and representativeness of the automatically generated bioethics ontology have been assessed by domain experts in general, and for pandemic bioethics with reference to COVID-19.

## Background

Ensuring that bioethics and the principles of ethics are positioned at the forefront and central to all day to day processes, related activities and actions, and intersecting sectors during pandemics is of paramount impact for many reasons. *Firstly*, it is well-known that vulnerable communities are most susceptible to the impact of a pandemic across sectors, including economy, health, education etc. Therefore, inequality of deployment of resources results in the suffering of these sectors and their communities. *Secondly*, during pandemics health personnel and scientists are actively developing therapies and preventive measures such as vaccines. Hence, it is more than often the case that vulnerable communities are taking advantage of to test new therapies and vaccines. For example, the history of clinical trials in Africa caused notable harm to people (5). Big pharma has a history of taking advantage of the lack of local policies and regulations to protect local citizens in many developing countries to come in and conduct vaccine and drug trials under the auspices of legal procedures. *Thirdly*, because of the development of technology and tracking systems to reduce the spread of a pandemic, people's privacy is being violated. Vulnerable communities—who do not have a voice or legal representation—are the ones who usually suffer the most.

Safeguard recommendations were introduced recently (6) such as data and privacy protection, where new technologies are used for surveillance in response to the COVID-19 pandemic. However, such technologies “may cause discrimination, be intrusive and infringe on privacy, or may be deployed against people or groups for purposes going far beyond the pandemic response” (6). Therefore, for these reasons collectively, bioethics principles and processes ought to be placed central to all governmental and civic society processes and sectors in response to pandemic operational spheres. In healthcare systems and society, McGuire et al. (7) discussed several ethical challenges in relation to healthcare systems and society such as informed consent and prioritization of healthcare workers. They found that multiple factors such as changing circumstances, experience, and patterns of illness play a role in reshaping ethical policy and reassessing ethical principles. They stress that learning from the COVID-19 experience is important for the next pandemic. On the same track, Saha et al. (8) indicated that professionals must be aware of the rapid change in the allocation of resources and evaluating healthcare standards. They also reflected on the technological impact in pandemics and stressed on the role of ethics to handle conflicts of interests and allocation of resources.

### Bioethics in a Process Context

Aksoy and Tenik (9) indicated that Bioethics is “a quasi-social science that offers solutions to the moral conflicts that arise in medical and biological science practice”. It is a systematic study of human conduct, which is interdisciplinary in nature within life sciences and healthcare, insofar as this conduct is examined in light of moral values and principles (10). The four principles of bioethics are: (1) “respect to autonomy,” (2) “non-maleficence,” (3) “beneficence,” and (4) “justice” (11). These principles govern the ethical conduct in almost every society. Bioethics links all healthcare professionals in an attempt to resolve ethical considerations for healthcare systems arising during patient care (12).

Healthcare systems comprise actors, processes, and activities in complex and dynamic environments with massive served and serving systems of systems interactions. However, these healthcare professionals require input from “multiple different disciplines, considering more than one perspective on the same phenomenon” (13). The adoption of a process centric approach in bioethics is of paramount importance in how information is gathered, and how relationships are managed between different stakeholders and systems involved (14).

It is observed that new directions have been emerging for theorizing about ethical decision-making and practice in healthcare contexts by drawing attention to new ethical actors, changing organizational settings with both broader ethical challenges and conceptualization of gate-keeping processes (15). Such emerging directions are becoming more orthogonal to healthcare services; and accordingly ethical review processes (16) will be in timely demand of data consumed and produced during the different activities of bioethics and healthcare processes. Such a requirement that necessitates building the ontology of the domain of bioethics.

### Bioethics in Ontological Context

One of the earliest definitions of ontology from a computing point of view is Gruber's definition “Ontology is a specification of a conceptualization” (17). A further more operationalized definition for ontology was provided by Noy and McGuinness (18) as “formal explicit description of concepts in a domain of discourse [classes (sometimes called concepts)], properties of each concept describing various features and attributes of the concept [slots (sometimes called roles or properties)], and restrictions on slots (facets (sometimes called role restrictions)).”

Several efforts have been put into integrating bioethics with ontologies. Koepsell et al. (19) developed the Biomedical Ethics Ontology (BMEO) as a methodology to guide the creation of “a powerful information tool”. The attempt was considered as “proof of concept”. However, DuBois (20) argued that such a framework was “ill-suited” for the entities related to regulatory definitions and ethical concepts. In addition, Wasilewska (21) evaluated the proposed BMEO framework to generate biomedical ethics ontology. He concluded that BMEO “might face unbeatable obstacles and the domain of moral consideration might not, at the same time, be an appropriate realm to be standardized by ontology tools”.

Recently Romanyshyn (3) attempted to show the importance of the ontological classifications and their relation to healthcare rationing. In general, his work set the common ground for the necessity of rationing especially with limited resources to ensure fairness between different parties from the same domain. However, he pointed out the need of relaxed range for accepting concepts “that would err on the side of generosity not facing hard choices”. He justified the importance of ontological classification in understanding psychological disorders. One of the main limitations of previous literature is the inability to produce a tangible ontology that can be used in practice. Also, no theoretical grounds for the concepts of bioethics (without any implementation), apparent comprehensive methodological research framework, and governing bioethics processes were observed.

Bioethics processes are heavily engaged in ensuring appropriate ethical conduct in relation to the associated healthcare systems and processes. Such ethical processes have data and information consumed and produced in relation to bioethics entities. Therefore, semantic heterogeneities are likely to emerge and new relationships are likely to proliferate between different entities, systems, standards, protocols, etc., that will dictate a complex governance requirement for the underlying bioethics processes. Consequently, this becomes very challenging in highly desperate context aware situations and with the massively changing context of dynamic environments such as the COVID-19 pandemic. Thus, in such complex and extremely timely demanding pandemic environments, ontologies are highly appropriate for resolving semantic heterogeneities at different levels of abstraction of bioethics and healthcare processes and systems.

In this research, we define “Bioethics Ontology” as *the structured and formal shared specification of bioethics concepts at different levels of abstraction along with the properties of these bioethics concepts, and the rules that govern the integrity of the relationships between them such that the specified principles and processes of bioethics are adhered to*.

## The iOntoBioethics Research Framework Design

In order to gain a comprehensive coverage of bioethics concepts and their evolution since they first appeared in the literature in 1971, we have developed a novel agile framework to mine the substantially impactful and well-indexed literature. This agile framework is empowered by fit-for-purpose Text Mining and Machine-Learning (TM&ML) engine that automatically identifies bioethics topics and their associated concepts. Such a framework needs to be agile to evolve with new changes or new topics and concepts emerging as new research, policies, legislations, quality and ethical requirements, etc., are published. Such intelligently generated bioethics topics and concepts are the key building blocks for our novel framework in its agility to evolve the construction and evolution of a universal bioethics domain ontology.

Furthermore, the iOntoBioethics framework adopts the Design Science Research Methodology (DSRM) (4) which enacts a problem-based solving paradigm for understanding, conducting, evaluating, and publishing this work. Given that the nature of the iOntoBioethics framework being a software engineering and information systems artifact, the DSRM methodological approach and its process are fit-for-purpose compared to other research methodological approaches that are more suited to laboratory or humanities research projects. The DSRM approach has been widely used and reported in the literature over the past years with notable examples (22, 23). Following the inception phases of problem formulation and objectives' definition, the DSRM process iteratively implements whole increments of design, development and evaluation activities during the whole life cycle of the research framework development before the final phase of communicating research project outcomes. This means that researchers can revisit and re-evaluate the developed framework as duly needed in order to tune the phased and final outcome in meeting the research aim and objectives (24).

In this research, the systematic literature mapping method has been adopted to address our research aim through the development of the iOntoBioethics research framework utilizing the DSRM. The DSRM fit-for-purpose process was devised with the incremental and iterative phases of design, implementation and evaluation before communicating outcomes in the final phase.

**Figure 1:**
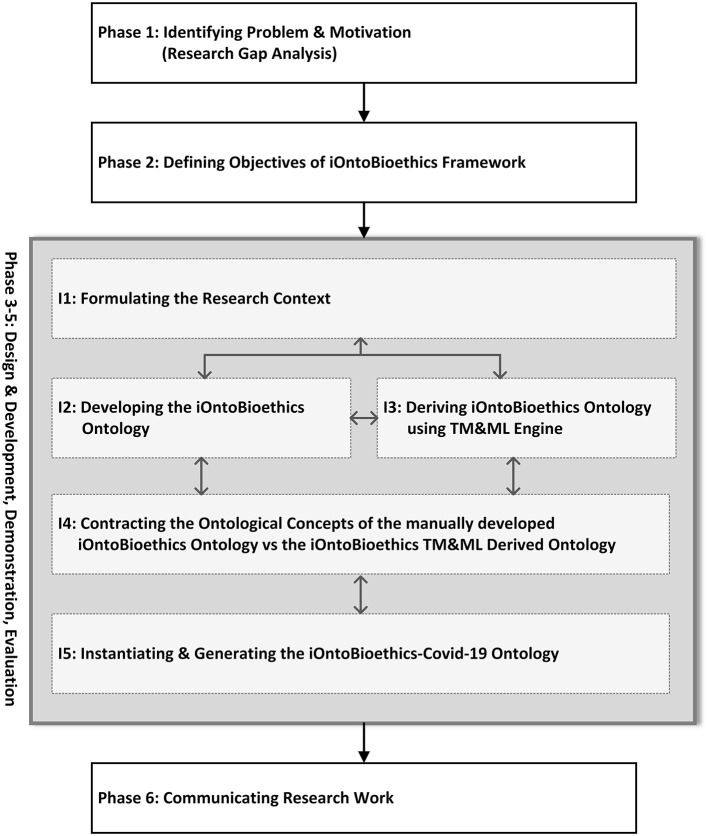
The iOntoBioethics research framework design.

The development of the iOntoBioethics framework has been carried out over five increments as shown in Figure 1. Although Figure 1 depicts linear stages of the iOntoBioethics DSRM process, some iterations and interleaving occur between this process increments from design to evaluation. Besides publishing this article and developing an open platform as a research outcome, as per developments published on the www.iOntoBioethics.org website. The website aims to involve the scientific community of researchers from different disciplines that are interested in collaborating their bioethics and/or ontology-related work in relation to this proposed agile framework.

### Phase 1: Defining the Research Problem—The Research Gap Analysis

In this phase, the research problem and rationale are identified. Based on the literature, a notable absence of a generic conceptualization model of bioethics domain is recognized, and in particular the absence of a model that operates in pandemics time.

### Phase 2: Define Aim and Objectives of the iOntoBioethics Ontology

The iOntoBioethics framework is agile and evolves with emerging research, policies, legislations, quality and ethical requirements, standards, etc. Therefore, this research aims to develop an agile, highly representative, and robust ontological model of the domain of bioethics in general, and amidst pandemics in particular such as COVID-19. This aim will be achieved when it assists in resolving semantic heterogeneities in the domain of bioethics that may arise because of the different uses of terms, processes, or standards. Therefore, the iOntoBioethics ontology becomes the central body that facilitates a standardized communication language in order to achieve better understanding of bioethics processes and in the automatic governance of these processes when linked to the respective information systems operating in healthcare centers, research and development institutions, civil society organizations, and businesses impacting or affected by bioethics. This phase was led by domain experts in the bioethics domain. Finally, the agility dimension of this framework is driven by a number of factors such as responding to agile changes to the domain of bioethics in relation to bioethics processes, standards, national legislations, technology evolution, etc.

Consequently, the iOntoBioethics research design has been orchestrated based on the following main research hypothesis “investigating the bioethics research literature, from the inception of bioethics research publications, leads to identifying a highly agile representative set of bioethics conceptual entities, and governance relationships of bioethics processes”. To assist in proving this hypothesis, the following two research questions were formulated:

*RQ1. How to capture bioethics ontological concepts highly holistically and align them with the COVID-19 pandemic in an agile form?*
*RQ2. How to evaluate the representativeness of these captured ontological concepts and their relationships within a bioethics COVID-19 ontology?*

### Phases 3–5: Design and Development, Demonstration, and Evaluation

This part of the iOntoBioethics framework was accomplished in five distinctive increments iterating over the three stages of the DSRM process: design and development, demonstration, and evaluation as depicted in Figure 1. Throughout these three phases, bioethics domain experts input and validation were taken. Each of these five increments yielded a significant part or artifact of the iOntoBioethics framework.

### The First Increment: Development of the Selection Process of Bioethics Research Sources

The systematic literature mapping method (25, 26) has been employed to guide the bioethics literature classification scheme and the bioethics research contents selection. Upon the formation of the research questions in DSRM phase 2, the well-known Scopus database was selected as the source of studies extracted. Scopus enabled the automatic importing of bibliographic data from scientific publications via the Scopus Database Application Programming Interface (API) (27). In addition, Scopus provides a more accurate representation compared to other databases in the area of bioethics and sciences (28).

The “bioethics” keyword was used as the search term to select the maximum set of bibliographic sources in relation to the field of study in this research without any time restriction. The search process was conducted in June 2020 using the Scopus API to ensure automation and accuracy, which resulted in 26,170 articles distributed over 5,045 sources originating since 1971. These articles established the base to drive advanced analysis of the bioethics literature in order to feed into the development of the iOntoBioethics ontology in two independent strands or increments: second increment and third increment, where the former is associated with the manual construction of the iOntoBioethics ontology and the latter adopting an automated special-purpose text mining and machine learning engine.

For the purpose of manually constructing the iOntoBioethics ontology in strand 2 or the second increment, further filtering and analysis of the 26,170 articles was carried out in order to arrive at a reasonable set of bioethics sources that can be rich enough to inform the identification of representative bioethics ontological elements. The formulated aim and objectives of these literature sources were used to manually drive bioethics ontological concepts. The selection process for this purpose implemented the following criteria and was carried out by the researchers and in conjunctions with the bioethics domain specialists:

(1) The source journals are Scopus indexed journals with maximum published number of articles related to “bioethics”;
(2) Involve the three-bioethics domain experts in an iterative process to identify the first 20 journals with the highest volume of articles related to bioethics;
(3) If the Scopus indexed journal is not in the list of the “top 100 bioethics journals” (29) and the 2019 Google List (30), other journals were screened manually by three domain experts and were added to the set of literature sources utilized in the manual construction of the iOntoBioethics ontology. Should the bioethics domain experts decide to remove any journal, they replaced it with journals that are common to both the Google Scholar 2019 list and the Hakkarinen list of 2015; and
(4) To gain better coverage of the bioethics domain, the research bioethics domain experts screened other journals related to the bioethics field and added them to the filtered set of literature sources. These were found to be rich with concepts related to bioethics and crossing over to pandemic bioethics.

The execution of the above criteria involved both machine and humans with quantitative and qualitative measurements. The machine provided fast retrieval of outputs that were then assessed with quality-based measurement by domain-experts to identify the journals that were missed by the automated search. As a result, the selected journals comprised nearly 25% of the total number of articles identified that were related to bioethics.

For the automatic generation of the ontological bioethics topic models and associated subjects, the full set of the 26,170 articles titles and abstracts were text mined and machine learned as explained in section The iOntoBioethics Research Framework Design and with the results in section results.

### The Second Increment: The Manual Construction of the iOntoBioethics Ontology

This increment is concerned with the manual construction of the iOntoBioethics ontology based on the filtered set of literature sources using the process and selection criteria described in phase one. First, the concepts that signify the scope of each journal are manually extracted and listed for the domain ontology modeler to utilize. Then, a preliminary concept map is generated and reviewed through a brainstorming activity with domain experts. Groups of related terms are arranged into top level classes then, incrementally, more classes are classified and arranged into a hierarchy. These ontological classes and the relationships between them are specified using the Ontology Web Language-Description Logic (OWL-DL) (31), First Order Logic decidable fragment (32). Using OWL-DL classifications are automatically computed, and any inconsistencies are detected. Protégé (33) has been used in this research as the ontology software development environment, which is an open ontology editor software developed by Stanford University. It supports OWL-DL, allows managing and reasoning the created hierarchies, and facilitates ontology graphical design and automatic validation. This paved the grounds for sharing bioethics common understandable knowledge representation agreed upon by bioethics stakeholders to reuse, and integrated with other domain ontologies as generally noted in Horrocks (32) and Kumar et al. (34). Bioethics domain experts evaluated the resultant manually constructed bioethics (MCB) ontology using the walkthrough approach of all the manually derived ontological concepts and their relationships.

### The Third Increment: The Automated Generation of the iOntoBioethics Ontology Using Text Mining and Machine Learning

The aim of this increment is to develop a special-purpose Text Mining and Machine Learning (TM&ML) engine that can be utilized to automatically discover bioethics ontological topics and related concepts using the titles and abstracts of the 26,170 bioethics research articles and the COVID-19 recent textbook of Kamp and Hoffmann (35). This textbook has been considered in this research for being a recent and highly comprehensive accumulation of the COVID-19 pandemic covered in the full chapters of *Epidemiology, Transmission, Virology, Immunology, Prevention, Diagnostic Tests and Procedures, Clinical Presentation, Treatment, Severe COVID, Comorbidities, Pediatrics, and Timeline*. Correlations between topics and their related concepts were observed and evaluated by the research bioethics domain experts. The output of this increment is composed of three artifacts: (1) the special purpose TM&ML bioethics engine, (2) agile, automatically generated, and evaluated topic/concepts generalized bioethics models enacting a generalized and automatically generated bioethics ontology, and (3) agile, automatically generated, and evaluated topics/concepts generalized COVID-19 models enacting a generalized and automatically generated COVID-19 ontology. Both of these enacted ontologies are further utilized in extending the generalized bioethics ontology to become the iOntoBioethics COVID-19 Ontology as the outcome of the fifth research framework increment.

### The Fourth Increment: Contrasting the Manually Constructed Bioethics Ontological Concepts to the Automatically Generated Ones Using the iOntoBioethics TM&ML Engine

This increment is aimed at contrasting the MCB ontological model to the TM&ML developed one, to inform agreement on common ontological entities, disagreements and which ontological elements have been missed in one and not in the other, along with domain experts consensus to yield the first validated iOntoBioethics ontology. Hence, the resultant bioethics ontological entities are assessed by both domain specialists and the ontology modelers *to inform the representativeness of the bioethics ontological entities including ontological entities for the governance of bioethics processes*.

### The Fifth Increment: Extending the Bioethics Ontology to Derive the iOntoBioethics COVID-19 Ontology

In this increment, the COVID-19 ontology generated in the second increment was utilized to extend the fully validated iOntoBioethics ontology in the fourth increment to become the finally constructed and validated Bioethics COVID-19 ontology, namely the iOntoBioethics first COVID-19 ontology. This final research artifact (or deliverable) marked the conclusion of the iOntoBioethics research framework implementation.

### Phase 6: Communication

The agile design and development of the iOntoBioethics ontology, and results from the cycles of phases 2–5 are incrementally communicated to selected bioethics domain experts and for publication in key healthcare and bioethics journals. In addition, it is aimed to publish the iOntoBioethics framework and its ontologies as an open platform to be utilized by informatics driven bioethics researchers, communities and healthcare centers and industrial platforms.

## Results

This section reports on the results of implementing the iOntoBioethics research framework with the incremental outcomes of developing the first agile, semi-automatically generated, literature-based, generalized, and domain experts validated two novel ontologies: (1) *bioethics ontology*, (2) *bioethics pandemic ontology in COVID-19 context*.

### The Manually Constructed iOntoBioethics Ontology (Second DSRM Increment)

The knowledge engineering methodology proposed by Noy, McGuinness and others (18) was adopted to manually develop the iOntoBioethics ontology. Though this methodology has been in existence since 2001, it naturally fits with the simple intuitive progression in ontology development whether machine interpreted or not. It is also one of the most commonly used methodologies for building research ontologies. It consists of seven iterative steps and suits small-scale ontologies. The work undertaken in each of these steps to manually construct the iOntoBioethics ontology is detailed below:

***Step 1:***The first task in this step is to decide the bioethics ontology's scope and boundaries. This depends on the domain of the ontology and the purpose for its use. As mentioned in section The iOntoBioethics Research Framework Design, the iOntoBioethics ontology aims to provide a general conceptualization model for bioethics that can be specialized for certain bioethics' spheres that may emerge and require special actions. Accordingly, the iOntoBioethics ontology's scope is determined to include all ethical issues related to medicine (and healthcare) and biology. In addition, disciplines, management activities, experiences, educational issues and religious issues related to bioethics have been included in the search space for bioethics ontological elements and associated relationships.

***Step 2:***This step recommends reusing existing ontologies instead of developing them from scratch. Therefore, ontologies can be imported and extended depending on the purpose for using them. In addition, ontologies can be imported and merged with other ontologies. A number of libraries of reusable ontologies are available on the web for these purposes. Reviewing the literature, it has been concluded that limited work is available concerning bioethics ontological conceptualization, specifically for generic ontological models that are capable of being instantiated for new situations such as the emergence of COVID19. Hence, the iOntoBioethics ontology was developed without any reuse of existing ontologies in order to fulfill this gap in the bioethics domain.

***Step 3:***In this step, key terms in the bioethics domain are enumerated. These bioethics terms were obtained from the scope of filtered set of journals following the systematic mapping literature review and the selection criteria detailed in section Phases 3–5: Design and Development, Demonstration, and Evaluation. These terms were enumerated in a list to eliminate redundancy. This process resulted in about 430 terms concerning bioethics, which formed the basis for the iOntoBioethics ontological conceptualization. Examples of such terms are: ethics, legal aspects, legislation, bioethics education, bioethics research, clinical practice, medical aspect, genetics, healthcare system, decision-making, etc.

**Figure 2:**
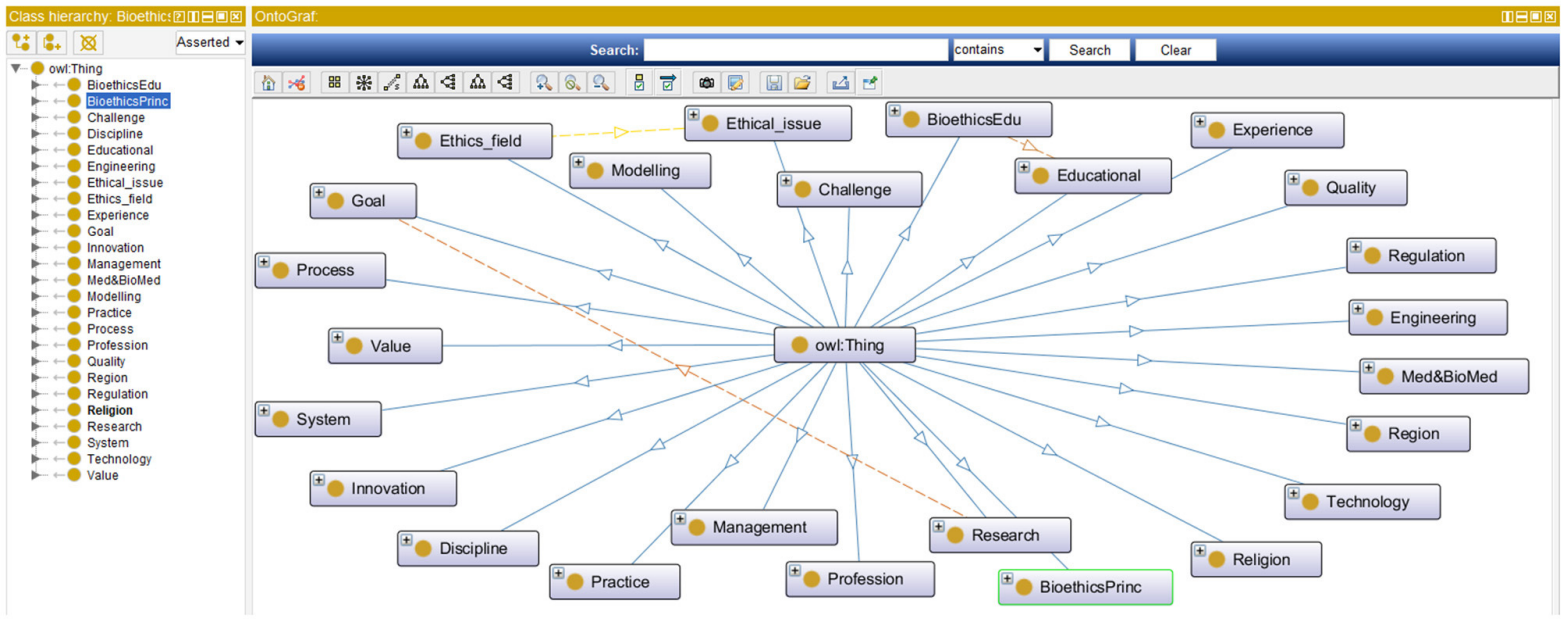
The top-level bioethics classes and the class hierarchy.

***Step 4:***In this step bioethics classes (or entities) are specified along with their bioethics class hierarchy. This step is intertwined with the previous one. While bioethics terms were collected, they were classified into meaningful bioethics groups to generate a bioethics concept map. Each group contained related concepts and was semantically linked to other bioethics groups. Class hierarchies can be developed either top-down, bottom-up, or a combination of both. Our approach in developing the bioethics class hierarchy for the iOntoBioethics ontology combined both top-down and bottom-up approaches. Each time a new bioethics term was encountered, it was placed either in one of the available bioethics groups if it was found appropriate; otherwise a new bioethics group was created, and then the concerned bioethics terms were either specialized or generalized according to the remaining available terms. For example, the terms “Aging,” “Animal human hybrids,” “Care,” “Cell topic,” “Clinical matter,” etc., can all be grouped into the top level class “Medical and Biomedical issue”. These terms can have more specific terms, for example all types of “Care” such as “Home care,” “Long term care,” “Community care,” “Complex care” etc., are added as subclasses to the “Care” class. The resultant bioethics class hierarchy consists of 25 top-level classes, and the remaining classes were in the middle and lower levels. Figure 2 shows the top-level bioethics classes and depicts part of the bioethics class hierarchy, both specified in OWL-DL and generated using Protégé (31).

**Figure 3:**
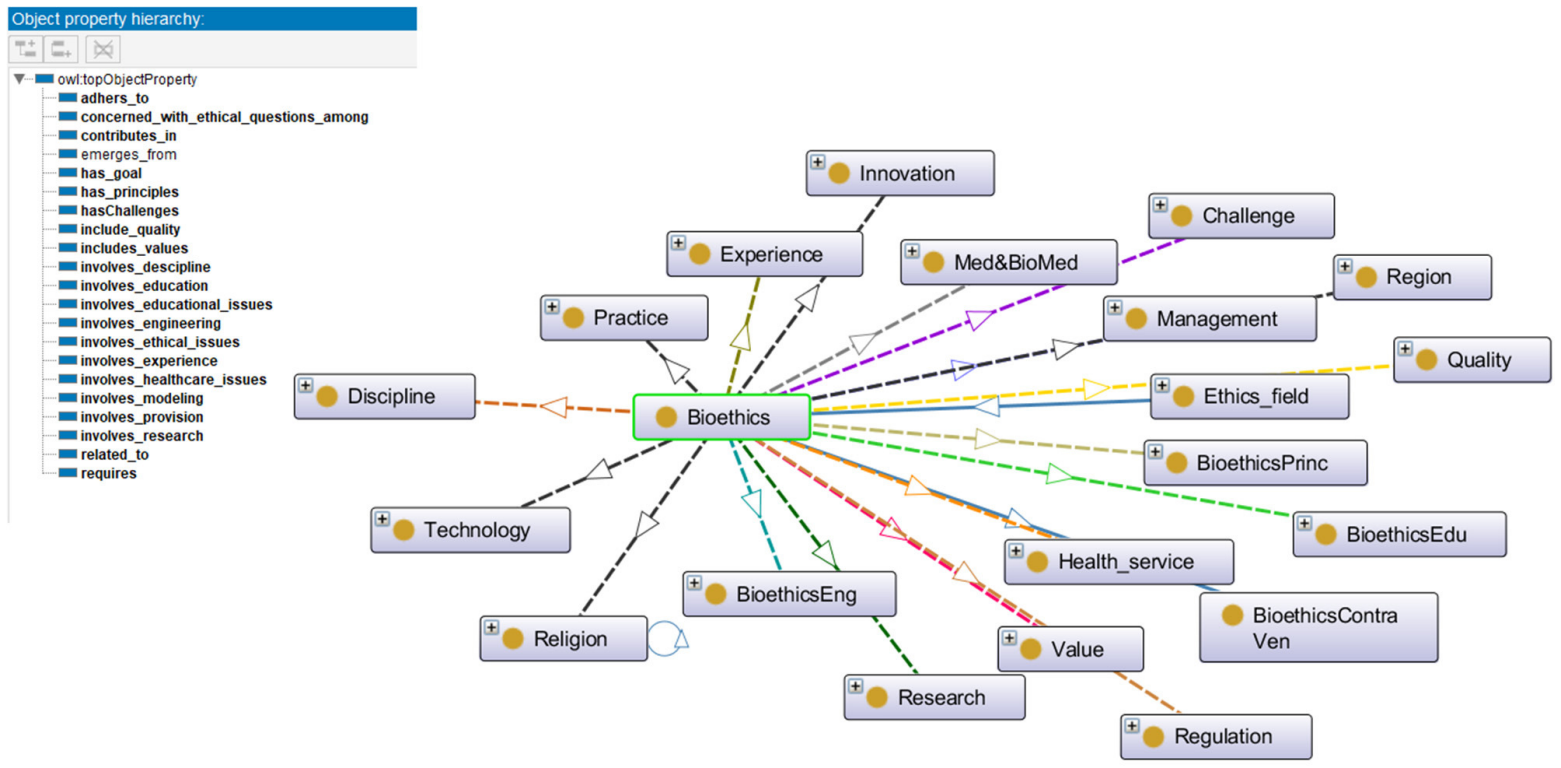
The properties for the Bioethics object, and the relationships between "Bioethics" class and other classes in the iOntoBioethics ontology.

***Step 5:***In this step the properties of the bioethics classes are identified and specified using the OWL-DL language. There are different types of properties: intrinsic, extrinsic, parts, and relationships to other individuals. According to the purpose of the iOntoBioethics ontology development, the aim is to represent the terms used in bioethics to assist in resolving semantic heterogeneities when interoperable in healthcare sector and especially *when interacting with related healthcare systems and Institution Review Board Systems* (36). Hence, the relationships between the bioethics classes need to be defined, and more specifically, the relationships between individuals of the class “bioethics” with all related top-level classes. With the domain experts' collaboration and guidance, 21 object properties were identified and specified as shown in Figure 3. For example, the property “adheres to” is defined to relate individuals of class “Bioethics” with those of class “Regulation and Legislation”.

***Step 6:***This is associated with defining features for the object properties, such as properties' domains and ranges, cardinalities, value types, etc. For each object property defined in the previous step for the iOntoBioethics ontology, the domain and range were specified. Class “Bioethics” is specified as the domain for most of the defined properties, such as “adheres to,” “has challenges,” “has principle,” “includes quality,” etc., and the ranges for the defined properties are specified, for example, the domain of the property “adheres to” is the class “Bioethics” and the range is the class “Regulation and Legislation”. Figure 3 shows the relationships between the “Bioethics” class and other classes in the ontology.

***Step 7:***This is the final step and is concerned with creating instances of bioethics classes. The iOntoBioethics ontology is a general and abstract ontological model that is used to conceptualize bioethics terms and set semantic relationships between them. This ontology can be instantiated for certain topics where individuals or instances can be created accordingly and operationalized for particular healthcare institutions and their systems, and now has the readiness for interacting with IRB systems and stakeholders.

### The Automatically Derived iOntoBioethics Ontology Using the TM&ML Engine (Third DSRM Increment)

One of the key motivations behind this research is that the bioethics research portfolio is rich in articles dating back to 1971. Thus, much of the hidden bioethics terms and relationships between them exist. Bioethics researchers, bioethicists, bioethics informaticians, and healthcare organizations can benefit from an automatically generated global or universal ontology of bioethics that can resolve semantic heterogeneities between bioethics concepts, terms, and associated. Such ontological construction facilitates interfacing to IRB healthcare systems and for developing bioethics semantic web applications with global software services that can be instantiated to inform adherence to bioethics processes governance in particular contexts, languages, cultures, legislations, etc.

Accordingly, the researchers hypothesized that an automatic generative process needs to be employed to generate ontological bioethics topics from the incrementally developing bioethics publications. These publications embed a hidden structure of bioethics topics that can agilely evolve with emerging publications added to the repository of bioethics publications. Hence, the goal of this automatic generative process is to discover these hidden bioethics topics and their underlying concepts from a repository of given bioethics publications. These underlying bioethics concepts relate to their certain bioethics topics with varying levels of statistical significance; and therefore, these bioethics topics relate to each of the bioethics publications with some statistical significance.

Accordingly, each of the given bioethics publications relates to the discovered bioethics topics but with varying proportions. It can be easily observed that we have two types of structures: observed and hidden. The observed structure is the bioethics publications, while the hidden structure relating to three key elements: (a) bioethics topics, (b) bioethics topics distribution per document, and (c) bioethics concepts assignment per bioethics topic in a bioethics publication. Consequently, such characterization fits with the motivation behind the Latent Dirichlet Allocation (LDA) (37, 38) algorithm in the field of machine learning. Hence, a *reverse engineering approach* is observed here, as we aim to discover the hidden structure (the bioethics topics and their associated concepts) from the observed structure (represented by the bioethics publications) in order to automatically discover our iOntoBioethics ontological elements and their associated concepts' relationships with varying statistical significance.

The agility of the iOntoBioethics framework stems from the unsupervised machine learning approach exhibited in our TM&ML engine that can dynamically reconfigure the bioethics topics vs. bioethics concepts vs. bioethics publications when implementing the LDA topic-modeling algorithm. However, the LDA algorithm requires as a precondition the known number of topics in the search and assignment space for topics vs. concepts. In this research, bioethics domain experts have been involved at the completion of this reverse engineering generative process, to characterize these LDA numbered topics with bioethics literal topics as discussed below.

However, before applying the LDA topic-modeling algorithm to the repository of bioethics publications, text mining had to be applied to the bioethics publications with a set of pre-processing steps applied to each of the collective text of these publications. The following process summarizes the implementation process of the fit-for-purpose TM&ML engine developed using R (39). This process was also reused for the automatic generation of ontological topic model of COVID-19 as discussed further on in this section:

1. *Studying the Bioethics Publications:*
  Following the completion of the first DSRM increment with the selection of the bioethics publications, the quality of the meta-data of these publications were checked for any anomalies such as duplication of entries, null values in their data attributes, etc. Notable examples were observed, for instance some of the publications did not have full abstracts included in the Scopus database;
2. *Consolidating the Bioethics Publications for Text Mining* (40)*:*
  In order to maximize the richness of the resultant bioethics' topic model, the textual volume of each of the publications is maximized to include publication title and abstracts to be text mined and machine learned using the LDA algorithm. This collective text for all bioethics publications is referred to as the *Bioethics Publications Texting Mining Database* (BPTM_db);
3. *Standardizing the BPTM_db Text Characteristics with reliance on R's TM (Text Mining) package* (40) *as follows:*
  - Convert all upper case characters to lowercase characters, so that all words in the text of each bioethics publication are in lower case.
  - Remove all stop words such as “the,” “on,” etc.
  - Remove all white spaces.
  - Remove all numbers.
  - Remove improper punctuations.
4. *Generate the Document Term Matrix (DTM) and apply the TF-IDF* (41, 42) *algorithm to normalize words or concepts occurrences amongst the bioethics publications in the BPTM_db:*
  - Perform the tokenization process, where each word in each of the BPTM_db publications becomes a token.
  - Construct the DTM and then TF-IDF matrix where the rows of the matrix represent the bioethics publication ids and the columns represent the tokens or words. Each row-column intersection provides the normalized count of the number of times a particular token or word has occurred in a particular publication.

Now, the DTM/TF-IDF matrix over the BPTM_db DTM is constructed for the bioethics publications. The LDA algorithm is applied to calculate the probabilities of the topics and their associated concepts or terms (words or tokens above) using Equation (1) from Blei et al. (37) and Blei (38):

(1) $$\begin{array}{r} p\left(\beta_{1:k},\;\theta_{1:D},\;Z_{1:D},\;W_{1:D}\right)\\ =\overset{k}{\underset{i=1}{\prod }}p\left(\beta_{i}\right)\overset{D}{\underset{d=1}{\prod }}p\left(\theta_{d}\right)\;\left(\overset{N}{\underset{n=1}{\prod }}p\left(Z_{d,n}|\theta_{d}\right)p\left(W_{d,n}|\beta_{1:k},Z_{d,n}\right)\right) \end{array}$$

where β_1:*k*_ represents the set of pre-input bioethics *k* number of topics, where 1 ≤ *i* ≤ k, and *k* is a pre-determined value, and each β_*i*_ is a distribution over words or concept in DTM, *q*_*d*_ is the bioethics topic proportions for publication *d*, q_d, k_ denotes the bioethics topic proportion the *k*^th^ topic in publication *d*, *Z*_*d*_ for the *d*^th^ publication topic assignments, *Z*_*d, n*_ denoting the *n*^th^ word topic assignment of publication *d, and* the observed words structure for each publication *d* is *w*_*d*_, such that *w*_*d*__, n_ is the *n*^th^ word of publication *d*.

The generative process for LDA corresponds to the following joint distribution of the hidden and observed variables, The conditional distribution of the hidden bioethics publications topics structure (and with associated terms or concepts) is called the LDA posterior probability computation adapted from Blei et al. (37) and Blei (38):

(2) $$\begin{array}{r} p\left(\beta_{1:k},{\;\theta }_{1:D},\;Z_{1:D}|W_{1:D}\right)=\;\frac{p\left({\beta_{1:k},\;{\;\theta }_{1:D},\;Z_{1:D},\;W}_{1:D}\right)}{p\left(W_{1:D}\right)} \end{array}$$

The joint distribution of the hidden bioethics topics structure is computed in the numerator of Equation (2), whereas denominator computes the probability of the observed structure of publications under a given bioethics topic structure.

**Figure 4:**
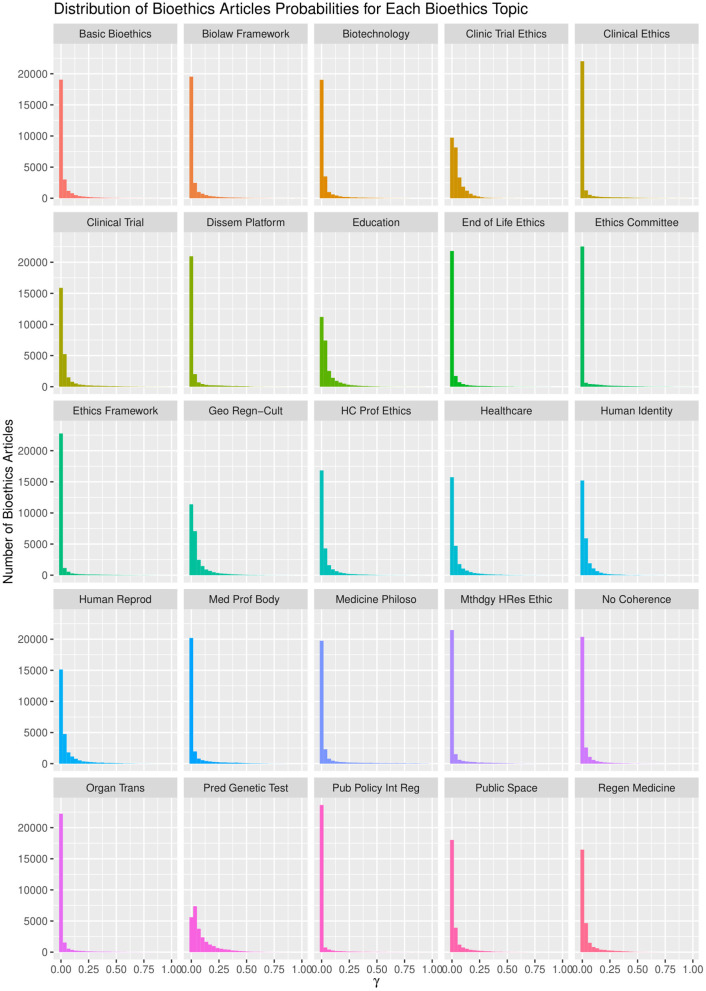
The 25 topics model and their associated information.

**Figure 5:**
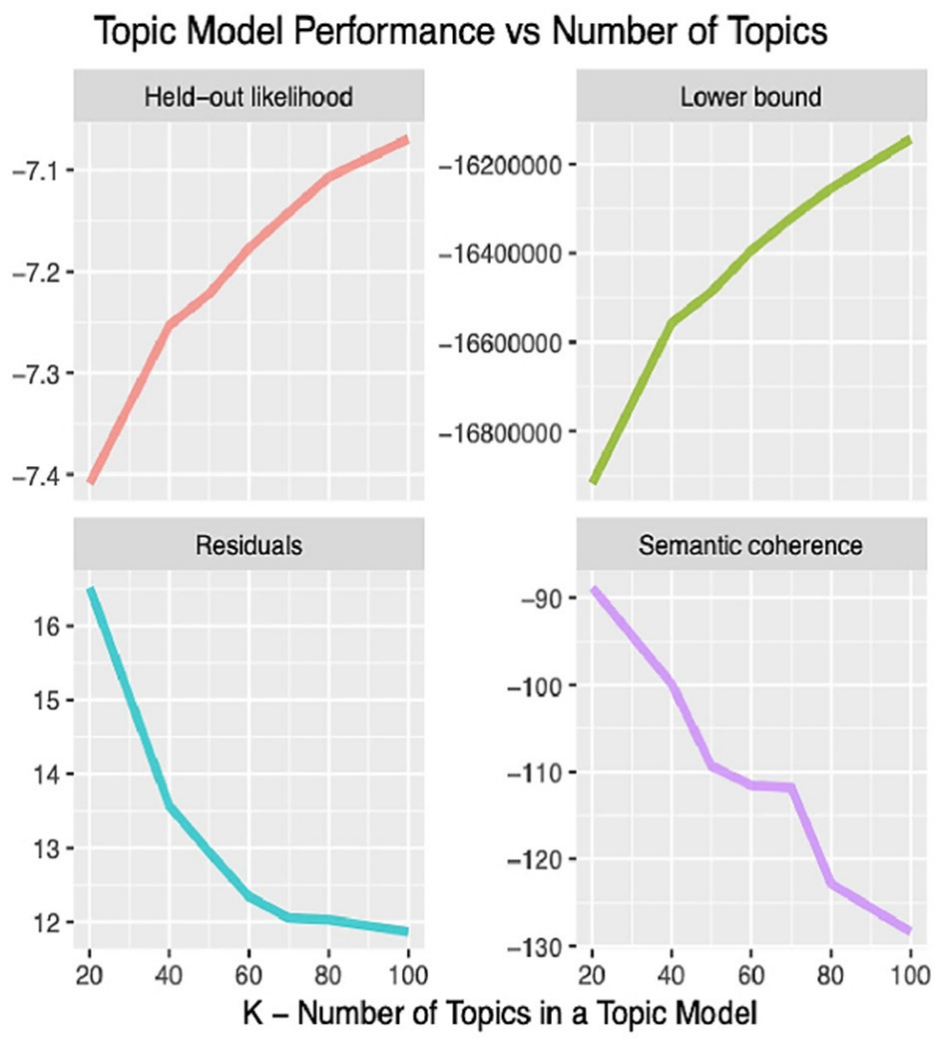
Topic model performance vs. numbers of topics.

The LDA algorithm topics model performance experimented with 20–100 bioethics topics. It was found that with the 40 topics model, the semantic coherence, holdout likelihood, lower bound, and residuals (43) had common performance measures as can be observed in Figure 4. However, it was found that after 25 topics, the concepts under these topics and the topics' themselves started to be redundant. In addition, this was found relatively coinciding with the number of core ontological bioethics classes of the MCB ontology in the second DSRM increment discussed in section The Manually Constructed iOntoBioethics Ontology (Second DSRM Increment). Figure 5 depicts the distribution of the bioethics publications for each bioethics topic, where similar probability distribution of bioethics across all the 25 topics is shown.

**Figure 6:**
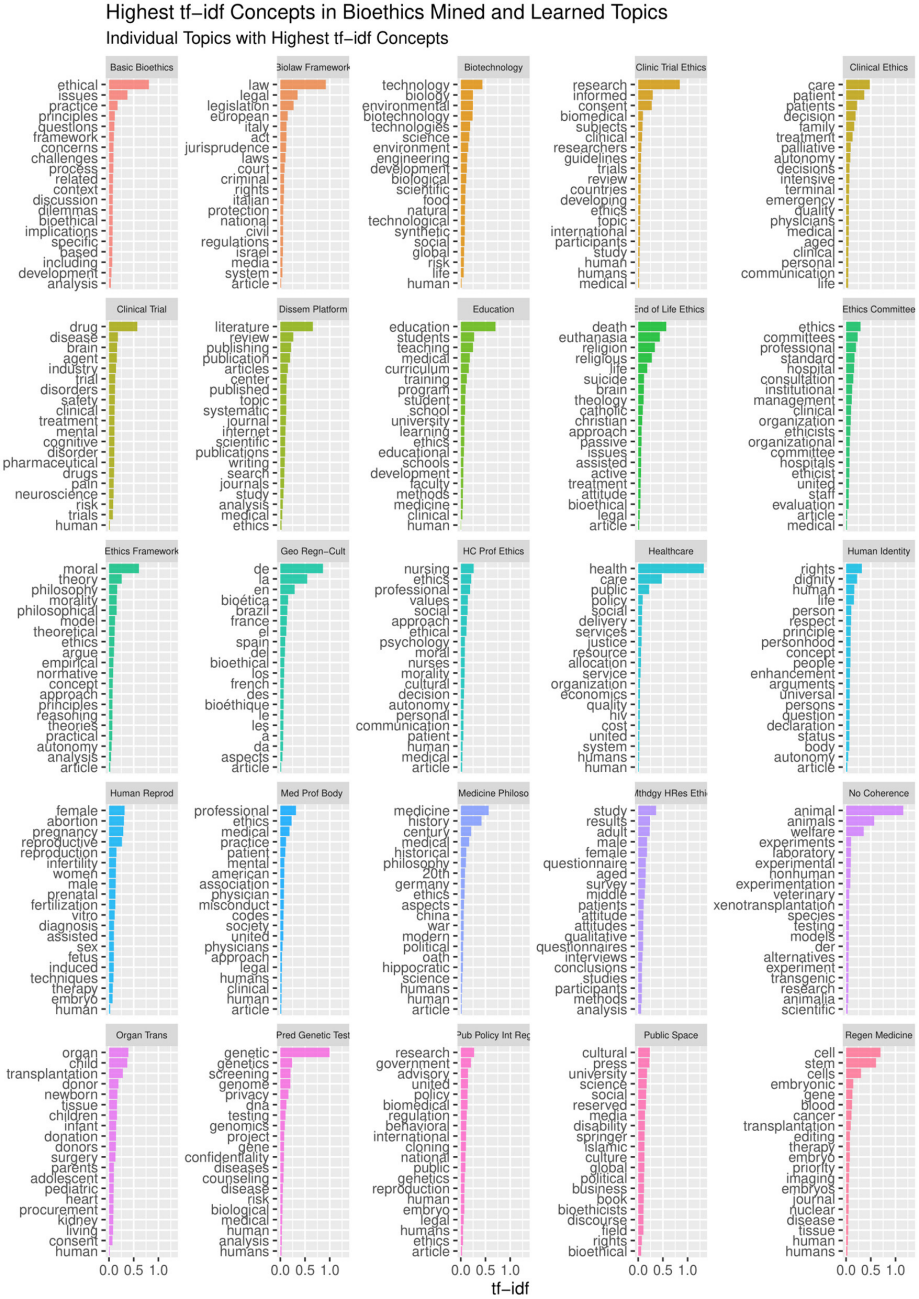
Each ontological topic with varying levels of statistical significance.

It is worth noting that the LDA algorithm does not name the topics discovered, but it assigns them random numbers within the range of the pre-defined number of topics. Therefore, we involved three bioethics domain specialists to study independently the 25 topic structures, arriving at a consensus of naming these 25 topics as depicted in Figure 6, with the concepts below each ontological topic with varying levels of statistical significance. These 25 topics represent the most significant topics automatically discovered using the LDA topic modeling with unsupervised learning in the first stage and then human-in-the-learning loop was deployed through these three bioethics domain specialists to arrive at this 25-topics model of the domain of bioethics along with the most significant 20 concepts per each of these topics. This 25-topics ontological model of bioethics was put forward for domain specialists to contrast against the MCB ontology as discussed in section The iOntoBioethics General Ontology—Domain Expert Validated (Fourth DSRM Increment).

**Figure 7:**
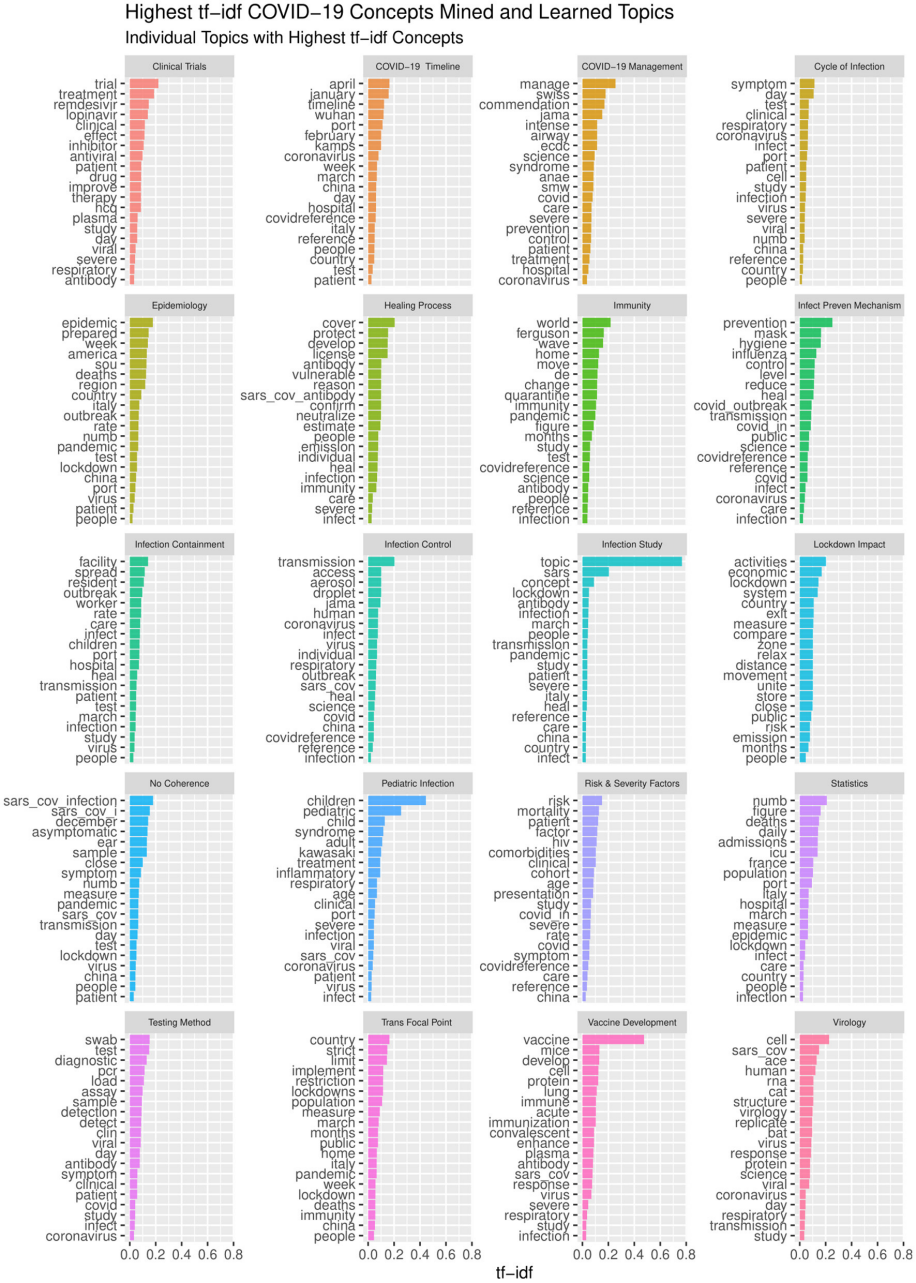
The highest 20 topics mined from the COVID-19 book.

The same TM&ML process applied to the bioethics publications was reused to generate the ontological topic model of the COVID-19 pandemic using the recent COVID-19 textbook of Kamp and Hoffmann (35) as discussed in section Phases 3-5: Design and Development, Demonstration, and Evaluation. Likewise, the bioethics ontology construction stages discussed above were re-adapted to apply the LDA topic-modeling algorithm to the full chapters of this textbook in order to automatically construct the COVID-19 ontology. Although the LDA topic-modeling performance was observed to saturate with semantic coherence around 40 topics, it was not found without redundancy after 20 topics, and hence the three domain specialists agreed on the naming of these automatically discovered as depicted in Figure 7. This LDA COVID-19 topics ontology has been used to link the iOntoBioethics merged and validated ontology in section The iOntoBioethics General Ontology—Domain Expert Validated (Fourth DSRM Increment) to yield the iOntoBioethics COVID-19 ontology, as discussed in section The iOntoBioethics COVID-19 Pandemic Ontology—Domain Expert Validated (Fifth DSRM Increment).

### The iOntoBioethics General Ontology—Domain Expert Validated (Fourth DSRM Increment)

**Figure 8:**
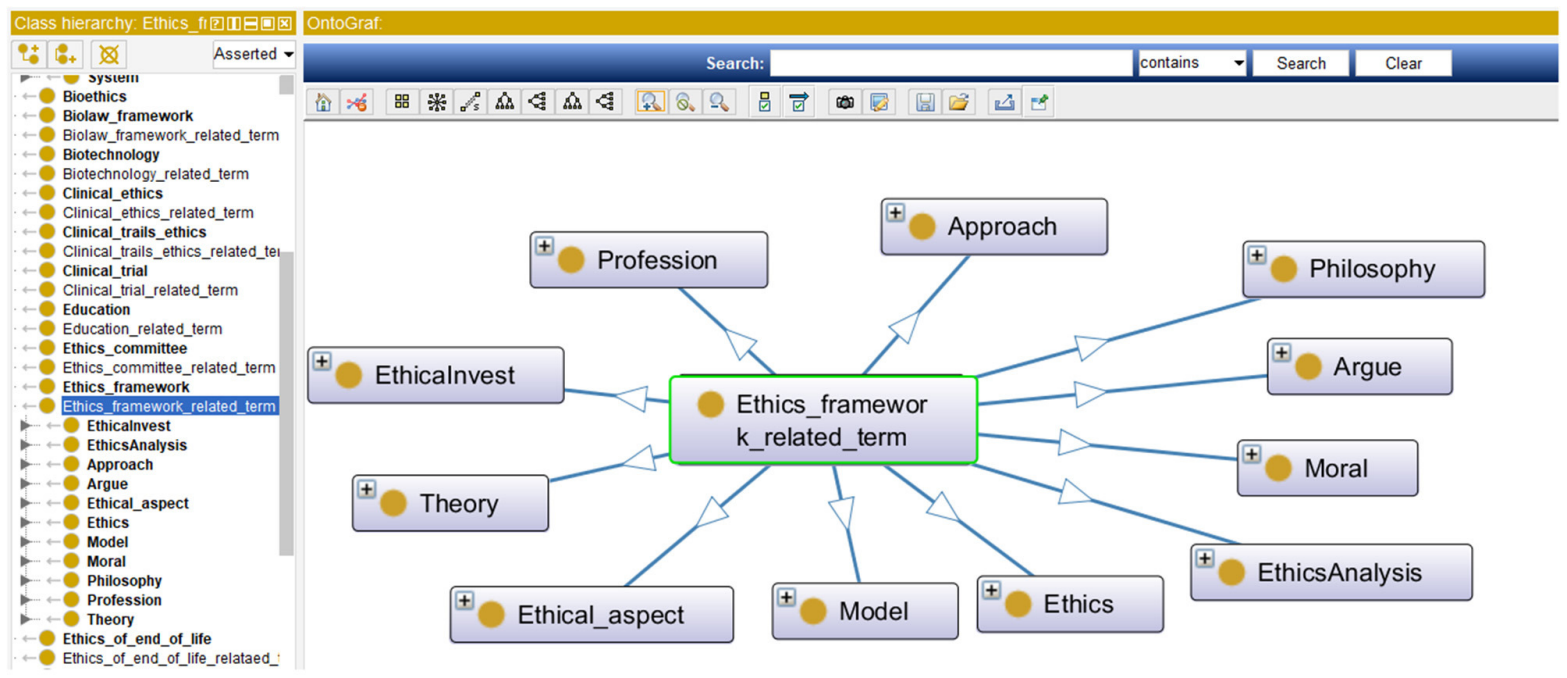
A sample ontological topics from MCB ontology.

In this section, the process for contrasting the MCB ontology and the TM&ML automatically generated one is described, followed by the outcomes of the finally agreed iOntoBioethics ontology. In general, the notable observation is that the TM&ML driven approach leads to deriving concepts at a higher level of abstraction and less specialization compared to the MCB one. For example, the topic “Ethics framework” that was generated from the TM&ML engine, is defined at a higher level of abstraction and at a lower level of specialization than the one generated from MCB. As shown in Figure 8, the detailed concepts from the MCB ontology reveals a different structure starting with the “Ethics” upper concept, which includes bioethics, model of ethics, and ethical issues as sub-concepts. The similarity of the main concepts exists in both ontologies with different structures as shown in Figure 8.

After investigating all the generated concepts from the TM&ML engine, eight topics were found to have similarities with the MCB ontology. The topics are: (1) *ethics framework, (2) medical professional bodies involved in human ethical issues, (3) education, (4) human identity, (5) geographical region, (6) predictive genetic testing, (7) platforms and channels for dissemination of ethical guidelines, and (8) Ethics of end of life*. Few topics (or classes) were unique to MCB, for example “animal ethics”. This distinguishes the MCB approach from the corresponding TM&ML approach. Few MCB topics (classes) were found to be more holistic than the corresponding concepts in the TM&ML ontology, such as the “Geographical Region” topic.

In addition, some TM&ML driven topics matched with the MCB topics (classes). These are: *(1) human reproduction, (2) human research ethics methodology, (3) human identity, (4) organ transplantation, and (5) clinical trials*. This is an indication of the substantial common ontological topics or classes that both the MCB and the TM&ML have been consistently in agreement with at a higher level of abstraction, and that the MCB approach yielded additional ontological; topics (or classes) at lower levels of abstraction such as “*clinical ethics*,” “*regenerative medicine*,” “*biotechnology*,” *and “professional healthcare ethics”*.

In addition, few topic concepts were found similar at their levels of abstraction in both approaches, yet having different details of the underlying classes such as the topic “*Public policies and international regulations”*. Also, it was observed that comparing topics and associated concepts, or properties of both the MCB and TM&ML bioethics ontologies did not always result in straightforward similarity between the topics and related concepts, for example 5 topics were similar and 19 others required subject interpretation to inform similarity consensus. Finally, one topic “*Clinical trials ethics”* of the TM&ML generated ontology matched a corresponding similar ontological topic in the MCB ontology.

Examining the TM&ML 25 generated ontology topics with the bioethics domains experts confirmed that the TM&ML approach provided a higher level of abstraction related to bioethics yielding a well-organized and structured bioethics ontology class hierarchy compared to the MCB bioethics ontology. However, the MCB based ontology provided more detailed and specific classifications of bioethics terms at lower levels of abstraction but with less structured class hierarchy.

**Figure 9:**
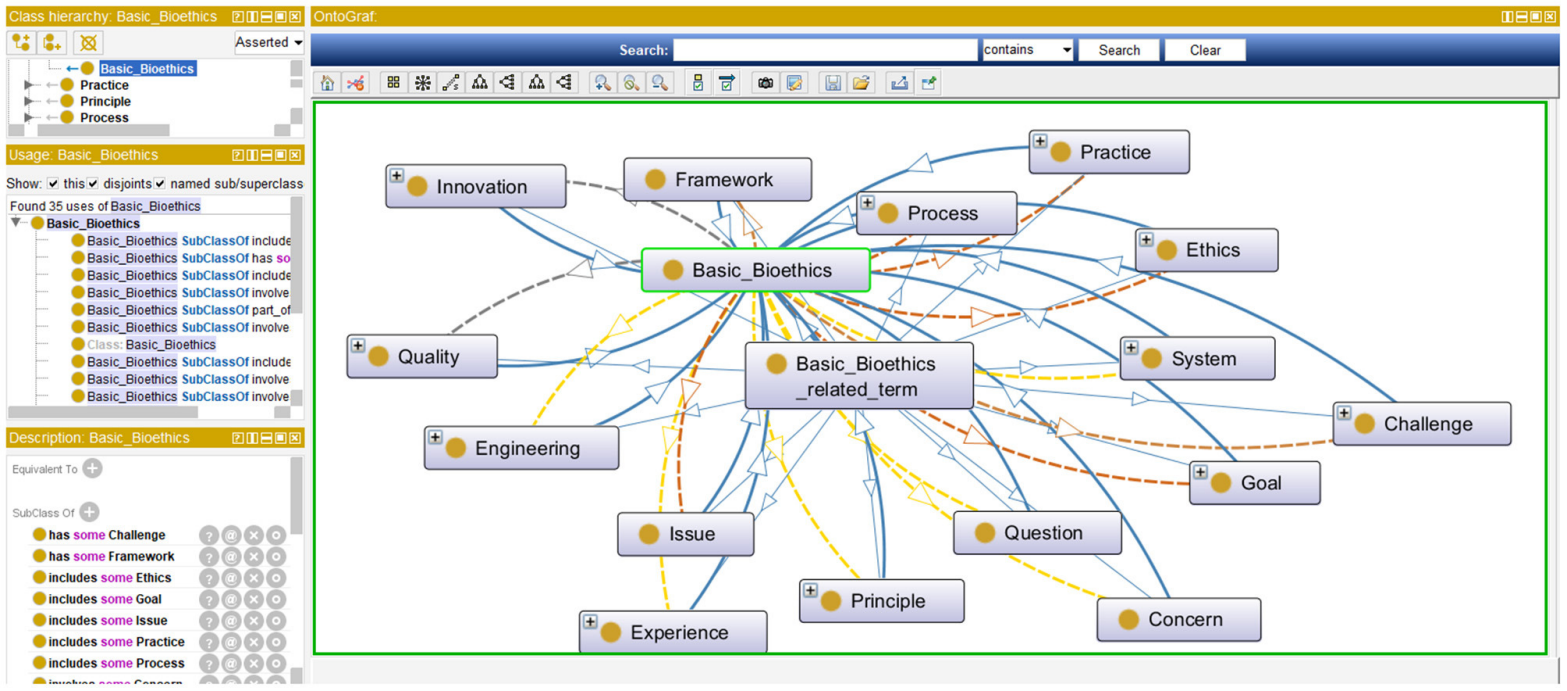
Part of the final bioethics ontology's class hierarchy.

Accordingly, both the MCB and TM&ML approaches complemented each other and that the MCB approach confirmed the findings and the ontological topics or classes and their related concepts and/or properties which resulted in a higher order unified and comprehensive bioethics ontology that can evolve with the incremental emergence of new bioethics literature. This higher order unified iOntoBioethics ontology contains 44 classes, 7 object class properties, and 697 SubClassOf class axioms with demonstration in Table 1. Considering the TM&ML 25 automatically generated topics (at higher level of abstraction) and the 20 concepts (at lower level of abstraction) as depicted in Figure 6 below each of these topics, it may be concluded that with these possible 25 × 20 (topic × concepts) relationships, variations between the MCB and TM&ML bioethics ontologies will continue to be the case, but most importantly the bioethics domain specialists confirmed the representativeness of the MCB and TM&ML ontologies in covering bioethics concepts and their associated relationships. Figure 9 depicts a snapshot of the iOntoBioethics ontology class hierarchy.

**Table 1:** The detailed MCB ontology classes that contributed to interfacing to the TM&ML ontology resulting with the iOntoBioethics unified bioethics ontology.

**Classes in the MCB ontology**	**Actions taken in the TM&ML ontology**
Bioethical principle	*Covered*—No action is needed
Bioethics education	*Class is added* (with all its subclasses) as a subclass of “Education”
Challenge	*Subclasses were added* to “Challenge” class under “Basic bioethics related term”
Discipline	*Subclasses are either covered or are irrelevant*—No Action needed
Educational issue	*Class is added* as subclass to “Education related term” with relationship “Education has some Educational issues”
Engineering	*Class is added* (with all its subclasses) as subclass of “Basic bioethics related term” with relationship “Basic bioethics involves some Engineering”
Ethical issue	*Subclasses were added* to “Ethics_framework_related_term” with relationships “involves some”
Ethics	*All subclasses are either covered in the derived ontology or are irrelevant*—No Action needed
Experience	*Class is added* (with all its subclasses) as a subclass of “Basic bioethics related term” with the relationship “Basic bioethics involves some Experience”
Goal	*Class is added* (with all its subclasses) as a subclass of “Basic bioethics related term” with the relationship “Basic bioethics includes some Goal”
Innovation	*Class is added* (with all its subclasses) as a subclass of “Basic bioethics related term” with the relationship “Basic bioethics related to some innovation”
Management activity	*Class is added* (with all its subclasses) as subclass of “Ethics committee related term” with relationship “Ethics committee involves some Management activity”
Medical_and_Biomedical_issue	*Most subclasses are either covered or are irrelevant*—no action is needed Subclasses of “Care” are added under “clinical ethics related term” “Cell topic” and its subclasses are added under “Regenerative medicine related term” with relationship “Regenerative medicine involves some Cell topic” “Drug issue” and its subclasses are added under “Clinical trial related term” with the relationship “Clinical trial involves some Drug issue.” “Gene related issue” and its subclasses are added under “Predictive_Genetic_testing_related_term” with the relationship “Predictive Genetic testing involves some Gene related issue.” “Health issue” and its subclasses are added under “Healthcare related term” with the relationship “Healthcare involves some Health issue.” “Healthcare issue” and its subclasses are added under “Healthcare related term” with the relationship “Healthcare involves some Healthcare issue.” “Illness” and its subclasses are added as a top level class with “concerned with” relationship to “Bioethics”
Modeling	*Subclasses were added* to “Model” class under “Ethics framework related term” class
Practice	*Subclasses were added* to “Practice” class under “Basic bioethics related term”
Process	*Subclasses were added* to “Process” class under “Basic bioethics related term”
Profession	*Class is added* (with all its subclasses) as a subclass of “Ethics framework related term” class with “involves” relationship to “Bioethics” class
Quality	*Class is added* (with all its subclasses) as subclass of “Basic Bioethics related term with the relationship “Basic Bioethics related to some Quality”
Region	*Subclasses were added* to “Geographical region” class
Regulation and legislation	*Subclasses were added* to “Public_Policies_and_international_regulations_related_term” class with “involves” relationship
Religion related issue	*Subclasses were added* to “Ethics of end of life related term” and with the relationship “includes”
Research	*Subclasses were added* to “Research” class under “ Clinical_trails_ethics_related_term” class
System	*Class is added* (with all its subclasses) as subclass of “Basic Bioethics related term” with the relationship “Basic Bioethics involves some System”
Technology	*Subclasses were added* to “Technology” class under “ Biotechnology related term” class
Value	*Subclasses were added* to “Value” class under “ Healthcare professional ethics related term” class

**Figure 10:**
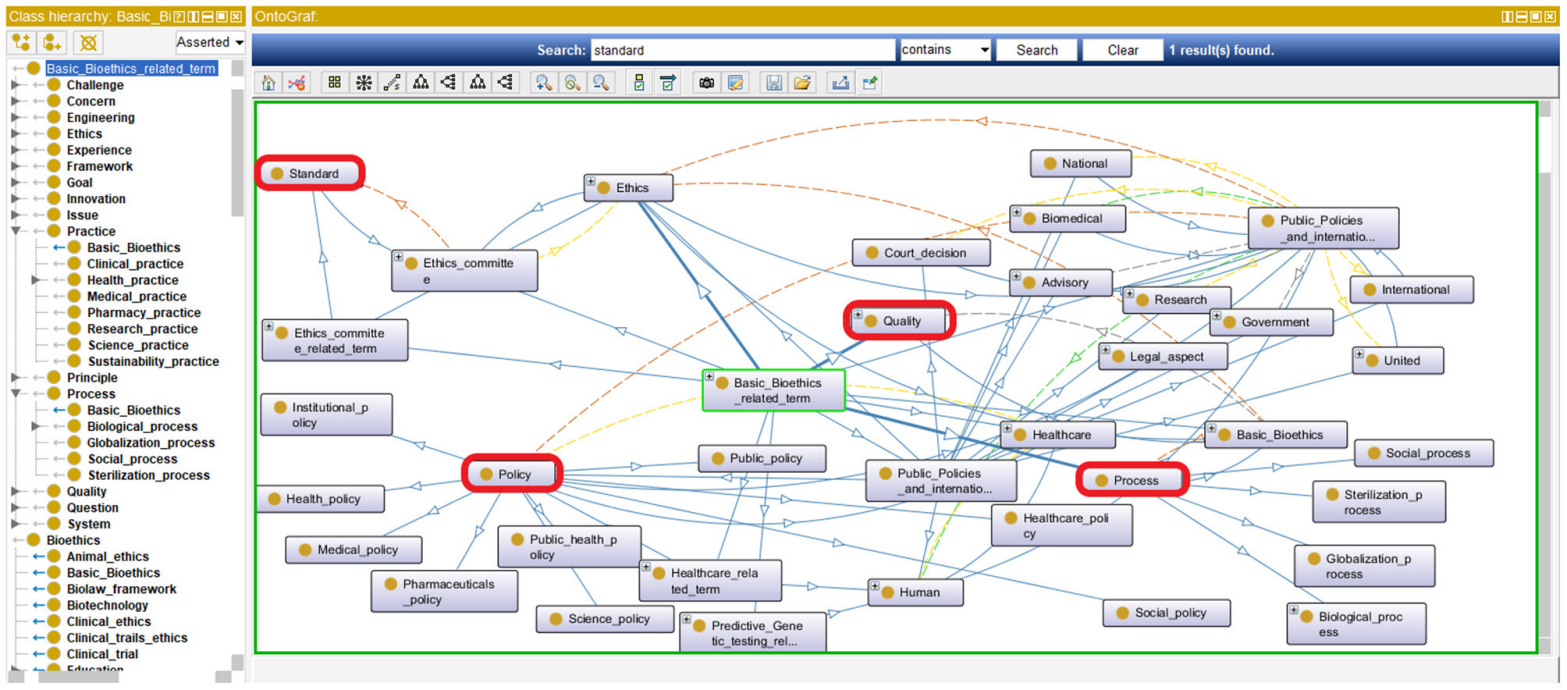
A Representation of the main governance quality attributes in the iOntoBioethics ontology.

The resulting iOntoBioethics ontology provides the concepts and semantic relationships that help achieve a better understanding of bioethics processes. In addition, it can be used to manage automatic governance of bioethics processes when linked to healthcare systems and research institutions. This can be seen from the representation of the four main governance quality attributes (44) highlighted in Figure 10 where the concepts: *policy, standard, process, and qua*lity are all specified and related in the iOntoBioethics ontology.

### The iOntoBioethics COVID-19 Pandemic Ontology—Domain Expert Validated (Fifth DSRM Increment)

The iOntoBioethics framework has been designed as a generic framework that when instantiated at any particular time using state of the art literature in bioethics, will semi-automatically generate a generalized bioethics ontology with validation by bioethics domain specialists. A first version of this generalized bioethics ontology was delivered by the completion of the fourth DSRM increment in section The iOntoBioethics COVID-19 Pandemic Ontology—Domain Expert Validated (Fifth DSRM Increment). In the fifth DSRM increment, this first version iOntoBioethics is utilized in a process centric approach to yield the iOntoBioethics COVID-19 pandemic ontology. This process is composed of the following steps:

(1) Create the iOntoBioethics COVID-19 Ontology as an empty container;
(2) Instantiate the iOntoBioethics ontology validated in section The iOntoBioethics General Ontology—Domain Expert Validated (Fourth DSRM Increment) to the iOntoBioethics COVDI-19 ontology container.
(3) Add a new ontology class named “Pandemic”.
(4) Create “COVID19” as a subclass of the “Pandemic” class.
(5) Walkthrough through the automatically generated COVID-19 topics using the special-purpose TM&ML engine in section The Manually Constructed iOntoBioethics Ontology (Second DSRM Increment). The bioethics domain specialists examined each of these 20 COVID-19 topics to inform its association with the bioethics domain. If a COVID-19 topic relates to the domain of bioethics, then it is added as a subclass of the COVID-19 class, otherwise this topic is ignored.

**Figure 11:**
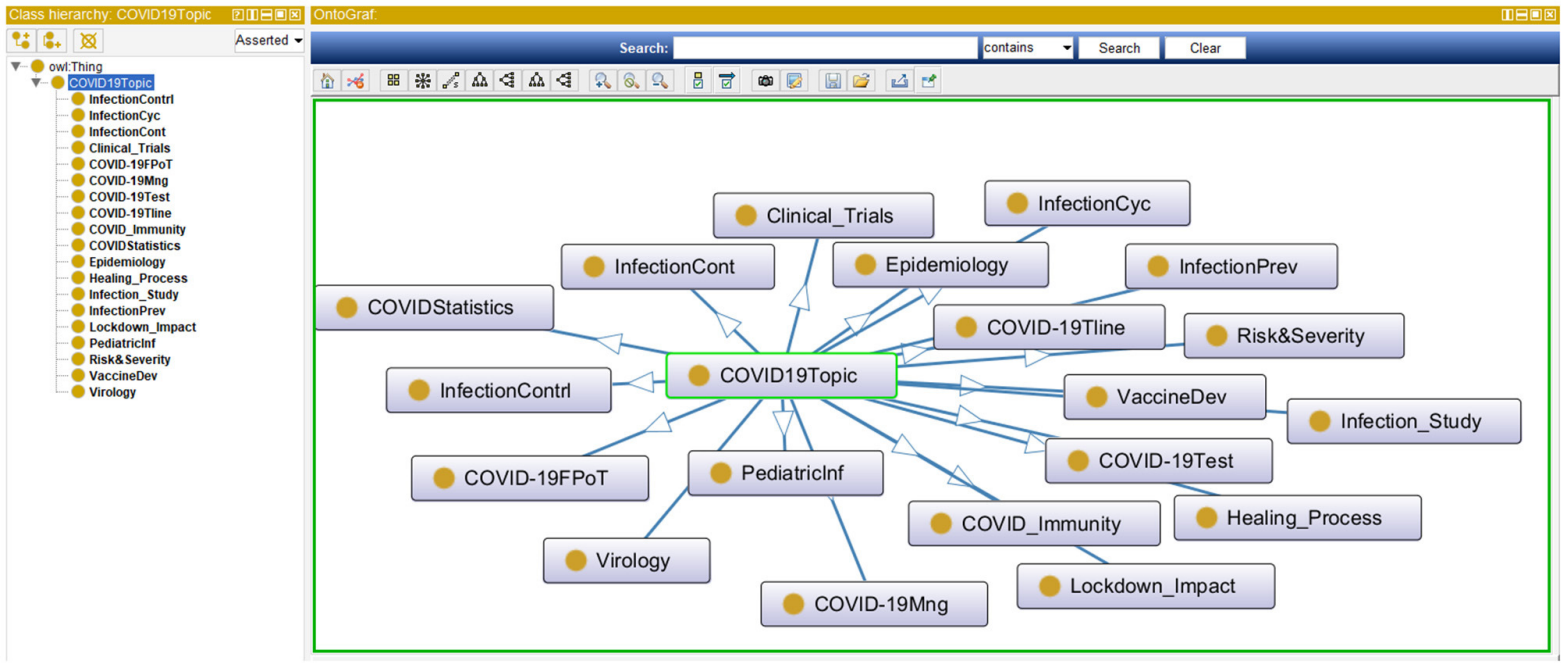
The COVID-19 ontological class model.

This process resulted in 19 classes integrated with the original iOntoBioethics ontology and jointly validated by the bioethics domain specialists to form the first unified novel Bioethics COVID-19 ontology as depicted in Figure 11 and detailed in Table 2. The primary linkages between the TM&ML COVID-19 topic model classes and the iOntoBioethics ontology are “*COVID19*” and the Bioethics classes, respectively. This “COVID19” class is linked to the top-level class “*COVID19 related topic*” through the “*involves topic*” class object property, and linked to the “*Bioethics”* class in the iOntoBioethics ontology through the “requires” object property. Finally, each COVID19 related topics' class was linked to the iOntoBioethics ontology's classes using their associated relationships. Algorithm 1 describes the above Bioethics COVID-19 process in general.

**Table 2:** COVID-19 TM&ML topic classes extending the iOntoBioethics ontology in forming the iOntoBioethics COVID19 ontology.

**TM&ML COVID19 topic classes**	***Actions taken to build the* iOntoBioethics COVID19 ontology**
Cycle of COVID-19 infection	*A new class is created with the relationship:* Part of “Predictive Genetic testing”
Healing process	*A new class is created as a subclass of* “Process” under “Basic Bioethics”
COVID statistics	*A new class is created as a subclass of* “Subject” under “Clinical trials ethics” and with the *relationship* part of “Research” under “Clinical trials ethics”
COVID immunity	*A new class is created with the relationship:* Part of “Research” under “Clinical trials ethics”
Lockdown impact	*A new class is created as a subclass of* “Challenge” under “Basic Bioethics”
COVID-19 management	*A new class is created as a subclass of* “Management” under “Ethics committee”
Vaccine development	*A new class is created as a subclass of* “Process” under “Basic Bioethics”
COVID-19 focal point of transmission	*A new class is created with the relationship: part of* “Process” class under “Basic Bioethics”
Infection prevention mechanism	*A new class is created with relationship:* Part of “Research” under “Clinical trials ethics”
Infection study	*A new class is created as a subclass of* “Subject” under “Clinical trials ethics”
COVID-19 testing method	*A new class is created with the relationship:* Part of Research
Infection containment	A new class is created as a *subclass of* “Process” under “Basic Bioethics”
Epidemiology	A new class is created as a *subclass of* “Subject” under “Clinical trials ethics”
Infection	A new class is created as a *subclass of* Illness
Risk and severity factor	*A new class is created for Severity Factor as a subclass of “Challenge” under “Basic Bioethics” (Risk is already covered)*
Infection control	*A new class is created as a subclass of “Goal” under “Basic Bioethics”*
Virology	*A new class is created as a subclass of “Subject” under “Clinical trials ethics”*
Clinical trial	*class is already covered*
COVID-19 timeline	*A new class is created with the relationship: Part of “Process” class under “Basic Bioethics”*

### iOntoBioethics Ontology Quantitative Evaluation

For ontology evaluation purposes, the metric-based ontology quality analysis OntoQA (45) was adapted. It is a feature-based method that utilizes the knowledge represented in the ontology to measure its quality. The features are divided into two groups to describe different aspects of the ontology: schema metrics and knowledgebase (instance) metrics.

Schema metrics evaluate the ontology design. They include relationship, inheritance, and attribute richness (AR). Relationship Richness (RR) shows the diversity of the relationships in the ontology, calculated as the percentage of the number of non-inheritance relations to the total number of relations, the higher the percentage is, the higher is the relationship richness. Inheritance richness (IR) indicates how good classes are grouped into categories; it is defined as the average number of subclasses per class, high IR means a horizontal ontology that covers wide range of knowledge with less details, while low IR indicates vertical ontology that covers only a specific knowledge area, but with more details. Attribute Richness (AR) is defined as the average number of attributes per class; this measure indicates the amount of information related to instances.

Knowledgebase metrics reflect the way data is placed in an ontology. These include class richness, class connectivity, class importance, cohesion, and relationship richness. Since our main research product is the abstract iOntoBioethics ontology—which is intended to be instantiated for specific bioethics domains—we only consider the schema metrics for evaluation purposes. Table 3 shows the results of evaluating the iOntoBioethics ontology as well as the two bioethics ontologies that were generated by the manual construction of concepts and by the TM&ML engine.

**Table 3:** Schema metrics results.

**Schema metric**	**Bioethics ontology (manual concept construction)**	**Bioethics ontology (TM&ML concept construction)**	**iOntoBioethics ontology**
Relationship richness	0.10	0.52	0.61
Inheritance richness	0.87	0.76	0.57
Attribute richness	0.06	10.7	0.6

As can be seen from Table 3, the average number of non-inheritance relationships per class in the MCB ontology was 0.1, while it was 0.52 using the TM&ML engine. As the iOntoBioethics ontology is an integrated composition of both ontologies (manually and TM&ML constructed), it was not surprising to have the highest relationship richness. Inheritance richness shows that the manually constructed ontology concepts appeared highly horizontal in the inheritance hierarchy, whereas the TM&ML-based ontology concepts are richer. However, the final iOntoBioethics ontology is the deeper ontology compared to the manually and TM&ML constructed ontologies. This reflects on the higher level of semantic enrichments that the integrated approach the iOntoBioethics ontology provides compared to either the manually and TM&ML based construction of ontologies. Finally, the AR metric shows poor number of attributes per class in the manually constructed ontology, richer in the TM&ML based ontology and neutral in the merged one, due to including more classes in the iOntoBioethics ontology.

**Algorithm 1:** Constructing the iOntoBioethics COVID-19 Ontology.

*# k: is the number of COVID-19 topics as can be traced to* *Figure 6**, which is 20 topics* *# l: is the number of COVID-19 topic concepts as can be traced to* *Figure 6**, which is 20 concepts*, *# for example the Cycle of Infection COVID-19 topic has 20 concepts such as day, test, respiratory, etc* # *iOntoBioethics COVID-19 Ontology* < –* iOntoBioethics Topics_Ontology* *For each topic*_*i*_ *in the TM&ML COVID-19 Topic Model (1*_≤_*i*_≤_*k), k* = *no of topics* *if topic*_*i*_ *does not exist in iOntoBioethics Topics_Ontology, then* *add topic*_*i*_ *as an ontology class to the iOntoBioethics COVID-19 Ontology* *endif* *for concept*_*j*_ *in topic i ((1*_≤_*i*_≤ *l*_*), l* = *no of concepts in topic i)* *if concept*_*j*_ *does not exist in iOntoBioethics Topics_Ontology, then* *add concept*_*j*_ *to the iOntoBioethics COVID-19 Ontology as an ontology class* *endif* *link concept*_*j*_ *to topic*_*i*_ *in the iOntoBioethics COVID-19 Ontology* *endfor concept*_*j*_ *endfor topic*_*i*_

## Discussion

In this section, we discuss the research we have conducted to prove the iOntoBioethics research hypothesis and its associated research questions bottom-up. This implies answering the two iOntoBioethics research questions first, and then reflectively concluding evidence to support the research hypothesis. In addition, we reflect on the effectiveness of the research design and the DSRM process adaptation in the development of the iOntoBioethics research framework design in achieving the main aim of this research. Finally, we conclude this section with reflections on the impact this framework is conjectured to have on the formal development of the new discipline we propose as “*Bioethics Informatics”* with reference to both agility, automation of governing bioethics processes in healthcare organizations, and the underlying software technology implications.

### Addressing the Research Hypothesis and Associated Research Questions

The hypothesis of this research states that “*investigating the bioethics and COVID-19 research literature, from the inception of bioethics research publications, leads to identifying a highly agile representative set of bioethics conceptual entities, and governance relationships of bioethics processes in general and COVID-19 in particular”*. In order to prove or disprove the hypothesis, the following research questions, RQ1 and RQ2 are answered first:

**RQ1:** *How to capture bioethics ontological concepts highly holistically and align them with the COVID-19 pandemic in an agile form?*
  The iOntoBioethics research framework has been designed with dedicated stages. First, well attributed and indexed bioethics research literature since 1971 until today have been captured through an automated open gateway (or API) to the Scopus (46) indexed literature database while applying the systematic literature mapping method (26) with domain expert validation of the automatically identified literature sources using a designated fit-for-purpose selection criteria as discussed in section The iOntoBioethics Research Framework Design. Therefore, the highly holistic (or comprehensive) dimension in RQ1 appears to have been well attended to with the resultant 26,170 literature sources.
  In addition, the agility dimension has been attended to through the special-purpose TM&ML engine that demonstrated effectiveness and representativeness in the automatic capturing of a set of bioethics topics (high level ontological classes) and their associated concepts (as either subclasses or associated ontological relationships) as briefly introduced in section The iOntoBioethics Research Framework Design and critically demonstrated in section Results using the LDA topic modeling machine learning algorithm (47). Also, a rich bioethics ontology has been manually constructed based on the literature selection criteria detailed in section The iOntoBioethics Research Framework Design and through applying ontology development methodology (31).
**RQ2:** *How to evaluate the representativeness of these captured ontological concepts and their relationships within a bioethics COVID-19 ontology?*

Three bioethics domain specialists have been incrementally engaged in the evaluation of the iOntoBioethics research framework artifacts (or products) as per the designated DSRM increments 2–4 detailed in section The iOntoBioethics Research Framework Design. First, the representativeness of the MCB ontology was manually assessed using the walkthrough software engineering validation technique (48) to validate the manually identified ontology classes and their relationships as discussed in section The Manually Constructed iOntoBioethics Ontology (Second DSRM Increment). This resulted in the first version of a manually bioethics ontology constructed from the scope of existing authenticated literature. This validated ontology has been cross-checked against the automatically generated TM&ML bioethics ontology using the research designated 26,170-bioethics literature sources. These two ontologies, the manually constructed and the automatically generated TM&ML ontologies were found to complement each other. Few bioethics ontological classes at higher level of abstraction were uniquely observed in the latter than in the former ontology. In addition, semantic heterogeneities between ontology terms, classes, associations have been resolved through the OWL-DL ontology language capabilities.

Both of these two bioethics ontologies have been cross-linked and cross-validated yielding the iOntoBioethics Ontology, *as the first enriched general bioethics ontology, agile-developed based on a profile of evolving authenticated and indexed literature*. Furthermore, COVID-19 ontological concepts have been automatically inferred through a designated recently published COVID-19 full textbook using the same TM&ML engine that has yielded automatically generated and generalized COVID-19 topics and their associated concepts. *These have been integrated to form the first semi-automatically generated, text-mined and machine learned Bioethics COVID-19 ontology using an agile process that can be re-instantiated to enrich the iOntoBioethics ontology as per the emergence of new bioethics and COVID-19 publications*.

Furthermore, cross-validating the linking of the iOntoBioethic generalized ontology and the COVID-19 ontological topic models by the designated bioethics domain experts was achieved through visiting every COVDI-19 ontological topic and its associated concepts, and assessing their proper association with the generalized iOntoBioethics ontology. *This has culminated in constructing the* first *Bioethics COVID-19 ontology within our framework that we have named the iOntoBioethics COVID-19 Ontology*. This will serve as an open universal platform to implement a full machine learning cycle, where the current bioethics publications served as the training data set, the newly emerging literature in bioethics and pandemics will be used as the testing dataset, to improve on and evolve the current state of the iOntoBioethics-Pandemic ontology.

The above attempt to answering the two research questions, RQ1 and RQ2, suggests that the research hypothesis has been answered with the following attributions:

(1) A highly generalized bioethics ontology has been constructed whose agility stems from the research framework design based on the special-purpose developed text-mining and machine learning engine that can be enriched, as per the evolution of availed authenticated and indexed bioethics and pandemic or COVID-19 literature;
(2) The iOntoBioethics generalized ontology (as per the last revised version of the evolving iOntoBioethics ontology) is proposed as a universal baseline to extend and specialize the bioethics domain within any potential healthcare challenges, illnesses, scientific revolution, or pandemics; and
(3) Consequently, related and specialized governance processes continue to be enriched as per the associated inner domain processes, quality requirements, standards, and policies as reflected on in section The Manually Constructed iOntoBioethics Ontology (Second DSRM Increment), contributing to the manifestation of these four aspects of governance.

### The Research Design Framework and the Impact of Adopting the DSRM Process

Adopting the DSRM process in the iOntoBioethics research framework design impacted the efficient undertaking and delivery of the research components and efficiently manage this research project with increased parallelism between project increments or tasks. For example, while the bioethics literature sources were being assessed by the bioethics domain experts, the development of the third DSRM increment of the “TM&ML engine” was taking place while the bioethics and COVID literature were availed. Also, the second and third framework DSRM increments continued in parallel in developing the manually constructed and automatic TM&ML bioethics ontologies. Such parallelism allowed some form of synchronization for the cross validation by the bioethics domain experts in the fourth and fifth DSRM increments, when both the iOntoBioethics general and the iOntoBioethics-Pandemic or COVID-19 ontologies were fully validated, respectively.

### The “Bioethics Informatics” Discipline and the Underlying Evolving Software Technology Implications

As ontologies play an important role in empowering Semantic Web Technologies (SWT) (32) and Internet of Things (IoT) (49), and hence they can be utilized to resolve semantic heterogeneities while exchanging knowledge for operating, managing, and governing bioethics operational and decision-making processes. As iOntoBioethics ontologies are developed using OWL-DL, and hence they are W3C's (32) Semantic Web compliant. Therefore, iOntoBioethics establishes an open platform for bioethics processes sharing new development in policies, regulations, legislations, e-consenting, standards, etc, to benefit big data analytics software services with enriched versions of multi-language and multi-culture support.

## Conclusion

**Figure 12:**
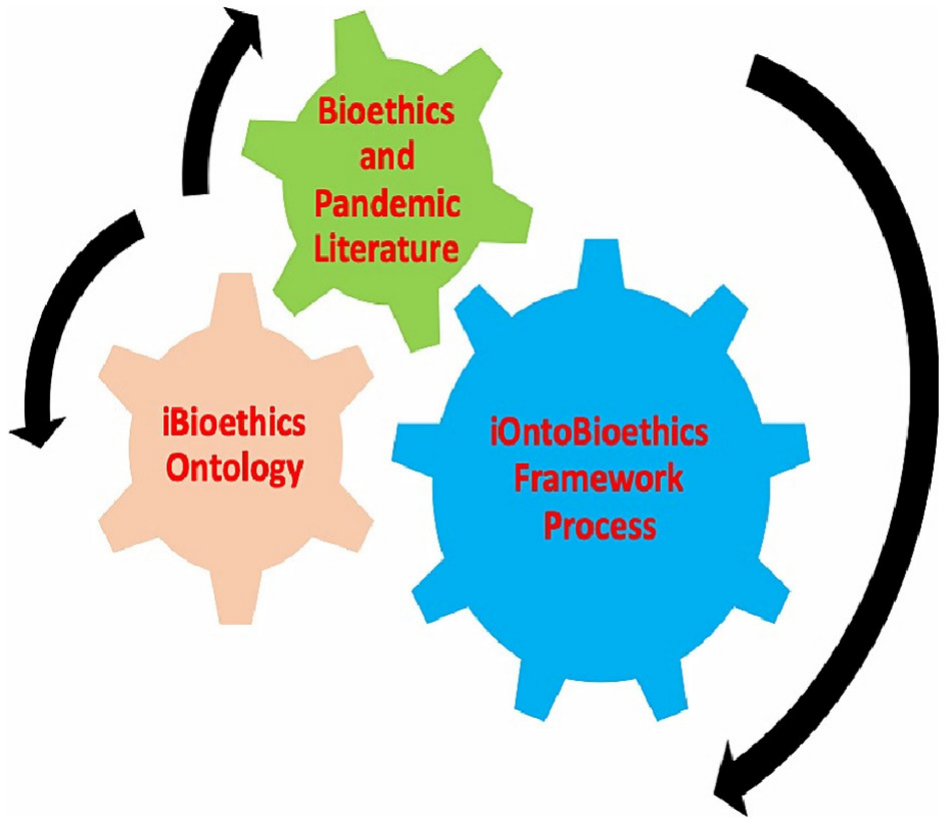
The Novel & Agile iOntoBioethics ontology construction process.

This research has been orchestrated with the aim to inform whether the current state of the bioethics and COVID-19 literature can be utilized for the agile development of a generic “Bioethics Ontology” that can be extended to a “Bioethics COVID-19” ontology aiding the automatic governance of bioethics processes in pandemics. The iOntoBioethics research framework has been developed adopting the Design Research Methodology with five fit-for-purpose cycles or increments that demonstrated both effectiveness and efficiency in achieving the research aim and objectives. This has resulted with the following four key novel artifacts (or products) for the bioethics research community and healthcare organizations:

(1) A generalized agile Bioethics Ontology, to serve as a common denominator to utilize and extend in particular healthcare contexts and settings;
(2) A generalized agile Bioethics COVID-19 Pandemic Ontology;
(3) The iOntoBioethics research framework with its agile process (depicted in Figure 12) that evolves with developing knowledge and literature in the field of bioethics and emerging pandemics or illnesses.; and
(4) An open platform for the (a) iOntoBioethics and (b) the iOntoBioethics COVID-19 Ontologies that is being hosted on the website for this research project with the URL: http://www.iOntoBioethics.org.

Furthermore, *the iOntoBioethics COVID-19 ontology has now emerged as the first publicized Bioethics Pandemic Ontology given the shared characterization of the COVID-19 ontology classes (or topics and associated concepts) with the generalized conceptualization of pandemics*. However, the scientific, healthcare and R&D communities, civic society and related organizations are still in their infancy stage of learning about COVID-19. Therefore, this first Bioethics Ontology will undergo a significant evolutionary wave, where the iOntoBioethics framework can agilely and semi-automatically evolve this ontology as per the process depicted in Figure 12.

Moreover, the iOntoBioethics ontologies can be extended to embed ontological conceptualization of specific metrics to assess *legal, social, ethical, and professional* adherence in healthcare organizations, regionally, etc. Finally, the iOntoBioethics framework establishes a foundation to linking bioethics processes and related healthcare systems to empower bioethics big data analytics.

## Data Availability Statement

The original contributions presented in the study are included in the article/supplementary material, further inquiries can be directed to the corresponding author and to https://www.iontobioethics.org.

## Author Contributions

Authors had various inputs such research framework design, sections writing with different levels of interest and expertise. MO research project leadership, research framework design, development of text mining and machine learning engine, domain and topic modeling validation, paper workflow, writing/co-writing sections of the paper, cross validation of ontologies, and final version review. FK research framework design, bioethics and ontologies review, contrasting of MCB and TM&ML ontologies, paper referencing, consolidation of sections, and final version preparation. RY research framework design, MCB ontology modeling, and extending to COVID-19. YO research framework design, ontologies cross validation, and referencing. DT research framework design, ontologies cross validation, and bioethics review. NH research framework design, ontologies cross validation, and bioethics review, bioethics from domain expert view. RD framework design, ontologies cross validation, and bioethics review, bioethics from domain expert view, and paper review. AM research framework, bioethics views, research project direction, and final paper review. All authors contributed to the article and approved the submitted version.

## Conflict of Interest

The authors declare that the research was conducted in the absence of any commercial or financial relationships that could be construed as a potential conflict of interest.
